# The effect of prepulse amplitude and timing on the perception of an electrotactile pulse

**DOI:** 10.3758/s13414-022-02597-x

**Published:** 2022-11-16

**Authors:** Jaspa D. Favero, Camilla Luck, Ottmar V. Lipp, Welber Marinovic

**Affiliations:** 1 School of Population Health, Curtin University, Perth, WA Australia; 2 School of Psychology and Counselling, Queensland University of Technology, Brisbane, QLD Australia

**Keywords:** Modularity of perception, Inhibition, Attention

## Abstract

The perceived intensity of an intense stimulus as well as the startle reflex it elicits can both be reduced when preceded by a weak stimulus (prepulse). Both phenomena are used to characterise the processes of sensory gating in clinical and non-clinical populations. The latter phenomenon, startle prepulse inhibition (PPI), is conceptualised as a measure of pre-attentive sensorimotor gating due to its observation at short latencies. In contrast, the former, prepulse inhibition of perceived stimulus intensity (PPIPSI), is believed to involve higher-order cognitive processes (e.g., attention), which require longer latencies. Although conceptually distinct, PPIPSI is often studied using parameters that elicit maximal PPI, likely limiting what we can learn about sensory gating’s influence on conscious perception. Here, we tested an array of stimulus onset asynchronies (SOAs; 0–602 ms) and prepulse intensities (0–3× perceptual threshold) to determine the time course and sensitivity to the intensity of electrotactile PPIPSI. Participants were required to compare an ‘unpleasant but not painful’ electric pulse to their left wrist that was presented alone with the same stimulus preceded by an electric prepulse, and report which pulse stimulus felt more intense. Using a 2× perceptual threshold prepulse, PPIPSI emerged as significant at SOAs from 162 to 602 ms. We conclude that evidence of electrotactile PPIPSI at SOAs of 162 ms or longer is consistent with gating of perception requiring higher-level processes, not measured by startle PPI. The possible role of attentional processes, stimuli intensity, modality-specific differences, and methods of investigating PPIPSI further are discussed.

## Introduction

### Sensory gating

Each day, our brains are tasked with navigating a complex and ever-changing environment. An environment filled with sensory information. This endeavour requires us to focus on relevant information while suppressing irrelevant information – a task that for most seems automatic and effortless. One mechanism contributing to the regulation of incoming information is called *sensory gating*. Sensory gating is not a singular process, but rather a set of neural processes that allow for or suppress the further processing of incoming sensory stimuli. In particular, here we were interested in understanding how sensory gating influences conscious perception.

### Sensory gating of reflexes: Prepulse inhibition (PPI)

Initially, sensory gating was examined in the context of lower-level stimulus processing by studying startle prepulse inhibition (PPI) – a phenomenon where the amplitude of a startle reflex elicited by an intense stimulus (known as the pulse) is reduced when shortly preceded by a weaker prepulse stimulus (Blumenthal, 2015; Graham, 1975). Due to the stimuli affecting a physiological response, PPI is considered an expression of sensorimotor gating (Blumenthal, 2015; Graham, 1975). PPI is highly sensitive to test parameters, particularly the time gap between the prepulse onset and pulse onset, known as the stimulus onset asynchrony (SOA). The phenomenon’s sensitivity to test parameters is useful in making inferences about the involved neural mechanisms, particularly its time-course of activation (Graham & Murray, 1977). Acoustic startle PPI, with both an acoustic prepulse and pulse stimulus, is the most extensively researched (Blumenthal, 2015). Within this modality, time-course studies show that PPI emerges at SOAs of 15–30 ms, peaking at around 60–240 ms SOAs (Blumenthal, 1999; Graham, 1975; Graham & Murray, 1977; Swerdlow et al., 2005). The shortness of the SOA required to observe PPI (15–30 ms) and the response that it acts upon (eye-blink reflex) have led to its mechanisms being proposed as pre-attentive (Blumenthal, 2015; Böhmelt et al., 1999; Swerdlow et al., 2005). That is, attentional mechanisms are unlikely to be involved at this level of sensory gating. This is further supported by the presence of PPIs in infants (Graham et al., 1981), sleeping adults (Silverstein et al., 1980) and even decorticated rats (Ison et al., 1991).

Human studies have found that explicitly directing participants’ attention to the prepulse, either by telling the participant to focus on or report identification that a prepulse was present, does enhance startle PPI. However, it has been extensively shown that this enhancement only begins to influence startle PPI at SOAs ≥ 120 ms, before which its effects are inconsistent (Ashare et al., 2007; Dawson et al., 1993; Elden & Flaten, 2002; Filion & Poje, 2003; Hawk et al., 2002; Heekeren et al., 2004). This suggests that prior to 120-ms SOAs attentional mechanisms are less involved, supporting that they are not a requirement for startle PPI, which is observed at SOAs of 15–30 ms.

### Sensory gating of perception: Prepulse inhibition of perceived stimulus intensity (PPIPSI)

In addition to the sensorimotor effects of PPI on the excitability of startle circuits, it has been subsequently demonstrated that PPI also modulates conscious perception – reflected by a reduction in perceived intensity of the intense stimulus (referred to as prepulse inhibition of perceived stimulus intensity, PPIPSI; Swerdlow et al., 2005). Initial theories suggest that PPIPSI and PPI are directly related, stating that the reduction in perceived intensity is a downstream effect of PPI on lower-level circuits (Blumenthal et al., 1996). For example, Blumenthal et al. (1996) proposed that participants’ perceived reduction in intensity (PPIPSI) is based on their perception of the reduced startle response (PPI), referred to as self-perceived startle. Consistent with the hypothesis that PPIPSI is a downstream effect of PPI are the findings of Blumenthal et al. (2001), where the perceived intensity of a subjectively painful electric shock (*M* = 160 V) was reduced by the presence of a weak prepulse (1× and 1.25× perceptual threshold) at SOAs of 40 and 60 ms, respectively. Observing PPIPSI at such short an SOA aligns with time-courses of preattentive lower-level mechanisms (Blumenthal et al., 2001). However, at high intensities, the separability of PPI and PPIPSI is limited by the fact that startle and subsequently PPI co-occur (Blumenthal et al., 2001). In fact, some studies have evidenced that startle is not a requirement for PPIPSI. At the trial level, significant PPIPSI is observed without the presence of startle PPI (Swerdlow et al., 2005) and using stimuli intensities sub-optimal for eliciting startle (Cohen et al., 1981; Peak, 1939). These and more detailed parametric experiments discussed below suggest that PPI and PPIPSI are related yet separable phenomena – such that PPI and PPIPSI are both driven by basic gating mechanisms at the lower level, but the manifestation of a perceivable reduction in intensity during PPIPSI is dependent on higher level (e.g., attentional) processes. This hypothesis is based on core findings of the strong correlation between PPI and PPIPSI when high-intensity stimuli are used, differences in their design requirements relating to directed/undirected attention and their time-course of activation (Swerdlow et al., 2005).

Detailed paradigm assessments by Swerdlow et al. (2005) investigated the effects of varying high-intensity acoustic stimuli (both prepulse and pulse) and SOAs on PPI and PPIPSI simultaneously. Both PPI and PPIPSI were found to be best elicited by higher intensity stimuli (80 dB prepulse and 105 dB pulse); under these conditions a strong positive correlation was observed (all *r* >.72; Swerdlow et al., 2005). This relationship suggests PPIPSI and PPI likely share underlying mechanisms. However, their results from manipulation of SOA (10, 20, 30, 60 and 120 ms) indicate a difference in the time-course of activation. PPI was observed from 30-ms+ SOAs, but PPIPSI was non-significant until 60-ms and maximal at 120-ms SOAs. This difference in time-course is consistent with conceptualisations of PPI as preattentive, and indicative that PPIPSI only occurs at temporal intervals that are sensitive to attentional manipulation (Swerdlow et al., 2005). PPIPSIs time-course also appears to align with when PPI becomes enhanced by directed attention (120 ms+; Ashare et al., 2007; Dawson et al., 1993; Elden & Flaten, 2002; Filion & Poje, 2003; Hawk et al., 2002; Heekeren et al., 2004). Consistent with the possible role of attentional mechanisms in PPIPSI, by design, PPIPSI requires cognitive appraisal of the pulse stimulus. This requirement is said to explicitly involve directed attention towards the pulse stimulus (Swerdlow et al., 2005). Conversely, PPI occurs under conditions widely considered preattentive (20–30 ms) and does not require the active engagement of the participant to be observed (Dawson et al., 1993; Graham et al., 1981; Ison et al., 1991; Silverstein et al., 1980).

These findings indicate that, while there is evidence supporting the association between PPI and PPIPSI, differences in the time-course of effects suggest that additional (likely attentional) mechanisms are involved for PPIPSI (Swerdlow et al., 2005). One hypothesis is that lower-level sensorimotor effects during PPI do contribute, but the degree to which the brain can access this lower-level information requires a top-down attentional shift toward the pulse. The observation of PPIPSI may therefore be shaped by time – the likelihood of observing PPIPSI may increase as SOAs become longer, as there is more time to orient to the pulse. Moreover, the observation of PPIPSI also appears to be shaped by prepulse intensity (Swerdlow et al., 2005), where the presentation of a more intense prepulse may facilitate the proactive shift of attention towards the prepulse-pulse pair.

However, one limitation of using intense, startle, and PPI eliciting stimuli is that they leave the degree to which PPI and PPIPSI are separable largely unknown. Independent studies have observed PPIPSI with sub-optimal startle intensities and stimuli modalities (Blumenthal et al., 1996; Cohen et al., 1981; Peak, 1939), but to date, the time-course of PPIPSI is only known using intense acoustic stimuli (Swerdlow et al., 2005).

### Current study

In the current study, we sought to characterise the nature of electrotactile PPIPSI by conducting three experiments examining how PPIPSI is influenced by parameters such as time (SOA between the prepulse and pulse) and intensity (of the prepulse). In Experiments 1 and 2, we examine PPIPSI under short and long SOAs. In Experiment 3, we explore the effect of prepulse intensity at 202-ms SOA where PPIPSI was prominent in Experiments 1 and 2.

## Method

### Participants

In all three experiments, participants were Curtin University undergraduate volunteers, who participated in exchange for course credit. We ran an a priori power simulation based on pilot data from five participants. This identified that for a repeated-measures generalised linear mixed model (GLMM) analysis with a power of 0.90 and α = .05, 19 participants were required. Consequently, a final sample of 25 participants (16 female, one non-binary) were recruited (age mean = 23.5 years, SD = 6.5, range = 18–49) for Experiment 1. An independent sample of 23 participants (12 female) volunteered for Experiment 2 (age mean = 22.8 years, *SD* = 5.9, range = 18–45). For Experiment 3, 24 participants (17 female) were recruited (age mean = 22.1 years, SD = 6.5, range = 18–46). All participants reported having normal or corrected-to-normal vision, with no known neurological conditions or injuries. In accordance with the Declaration of Helsinki and with approval from the Curtin University human research ethics committee, prior to participation, informed written consent was provided by all participants.

### Experimental task and stimuli

#### Experiment 1

Participants were seated at a desk with their head ~57 cm away from a 24-in. BenQ LCD monitor (1,920 × 1,080 resolution; 120-Hz refresh rate), with their arms rested on the desk. Two Digitimer DS7A stimulators (separate stimulators to deliver to pulse and prepulse) were then attached to the participants’ left wrist at the ulnar styloid process using four Kendall Covidien Ag-AgCl adhesive electrodes. Both stimulators were set to emit a single square wave prepulse or pulse with a duration of 2 ms.

Following this, a perceptual threshold (i.e., the weakest identifiable stimulation) was identified using a work-down and work-up procedure. Stimulation started at 0.50 mA and decreased in increments of 0.10 mA until the participant no longer reported feeling the stimulus. The intensity was then increased using finer increments of 0.05 mA until the stimulus was first perceived again – this intensity was defined as the perceptual threshold. Prepulse intensity was set to two times the perceptual threshold (e.g., 0.50 mA perceptual threshold = 1.0 mA prepulse intensity). Pulse intensity was determined by a work-up procedure with stimulation starting at the perceptual threshold and increasing in 1.0 mA increments until the stimulus was reported to be “unpleasant, but not painful”. Descriptive statistics of participants’ perceptual thresholds, prepulse and pulse test intensities are provided in Table 1.

**Table 1:** Means, standard deviations and ranges for stimuli intensities in Experiments 1, 2 and 3

Stimulus	Experiment 1		Experiment 2		Experiment 3	
	*M (SD)*	Range	*M (SD)*	Range	*M (SD)*	Range
Threshold	0.62 (0.44)	0.20–1.75	1.20 (1.01)	0.3–1.75	0.25(0.11)	0.10–0.5
Prepulse	1.24 (0.89)	0.4–3.5	1.24 (0.89)	0.6–3.5	*	*
Pulse	6.64 (3.55)	2–15	6.26 (6.17)	1–20	5.81(2.46)	1–20

*Note.* Unit of measurement = milliamps (mA). Prepulse intensity was manipulated in Experiment 3, thus descriptive statistics were 1, 2 and 3× the perceptual threshold*

To familiarise participants with the task, four practice trials were administered. Each trial contained two stimulus presentations: pulse stimulus alone (referred to as control) and pulse stimulus preceded by a prepulse (referred to as ‘pulse with prepulse’) presented at one of six different SOAs (0, 42, 82, 122, 162 or 202 ms). Each SOA configuration for the pulse with prepulse stimulus was presented 30 times (total number of trials = 180). On 50% of trials, the pulse-alone was delivered first. The order of stimulus presentation (i.e., pulse-alone or pulse with prepulse first) and the SOA condition were randomised. Within each trial, the time interval between the first (S1) and second (S2) pulse was randomised to 2, 4 or 6 s. Two seconds after S2 was delivered, participants were prompted to select via mouse clicking: “which shock-stimulus was perceived as more intense (left-click = first stimulus, right-click = second stimulus or middle-click = felt the same)?” Time between responding to the present trial and commencement of the next was also randomised to 1, 2 or 3 s.

#### Experiment 2

The same equipment and procedure were used as in Experiment 1, with the only changes being the SOAs investigated (0, 202, 302, 402, 502 and 602 ms).

#### Experiment 3

The equipment and procedure used were the same as those in Experiment 1, with the following exceptions. Three different prepulse intensities were investigated: one, two and three times the perceptual threshold. Participants completed three blocks of 40 trials each, with a different prepulse intensity for each block. Block order was counterbalanced between participants. A 202-ms SOA between prepulse and pulse was used throughout the experiment. The results of Experiment 1 indicated that PPIPSI was most prominent at 200 ms, and Experiment 2 showed that this was followed by a plateau. Although PPIPSI at 402-ms SOA was slightly elevated compared to 202 ms, we selected 202 ms because this interval coincides most closely with the shorter SOAs typically examined in the PPI protocols.

### Statistical analysis

All statistical analyses were conducted using R statistics (v3.5.1; R Foundation for Statistical Computing, Vienna, Austria). We conducted GLMM analyses using a logistic regression to model the proportion of prepulse-pulse trials perceived less intense, with SOA as a fixed-effect predictor (for Experiments 1 and 2) or prepulse intensity (for Experiment 3), with participant ID as the random factor. The GLMMs were conducted at the trial level using the ‘gamljGlmMixed’ function of the ‘gamlj’ package (Gallucci, 2019). To facilitate the interpretation of the data, we excluded ‘unbiased’ trials where the participant responded “felt the same”. Descriptive statistics of the excluded ‘unbiased’ trials for each experiment are provided in Table 2. Given the binary nature of the outcome variable, we used a binomial family distribution for the model. Follow-up pairwise comparisons with Holm’s adjustment for multiple comparisons and estimated marginal means for plots were extracted from the model output provided by the ‘gamlj’ R package.

**Table 2:** Mean percentage and standard deviation of unbiased trials removed for each experiment

Experiment no.	% Unbiased responses removed
	*M (SD)*
Experiment 1	30.20 (14.69)
Experiment 2	35.00 (16.65)
Experiment 3	21.64 (11.10)

## Results

### Experiment 1: 42- to 202-ms stimulus onset asynchronies (SOAs)

**Fig. 1:**
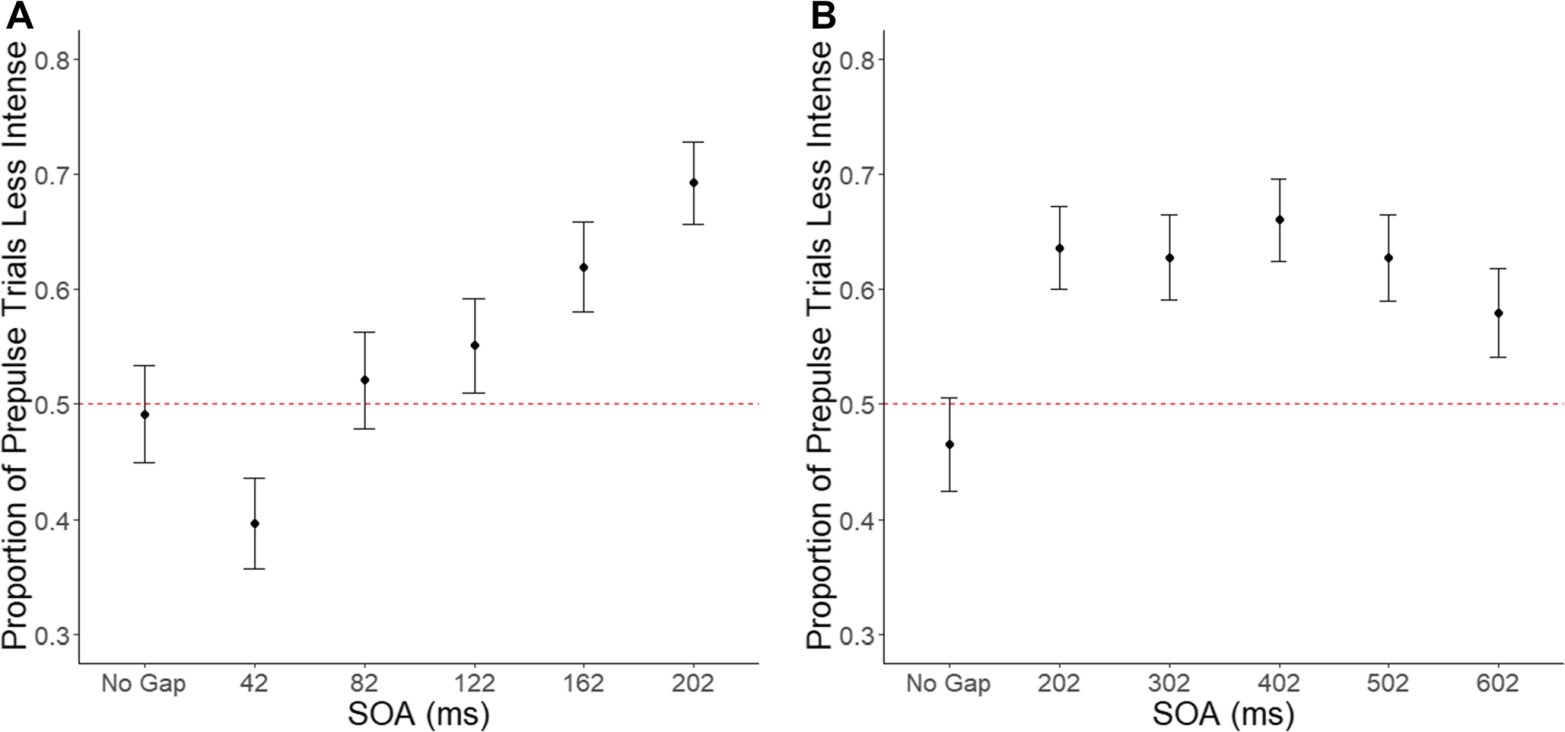
Estimated marginal mean proportion of trials perceived less intense for each stimulus onset asynchrony (SOA) investigated in Experiments 1A and 2B. The red dotted line represents chance level. Bars represent the standard error (SE) for each condition. 1A shows that as SOA increased, presence of the gating mechanism (PPIPSI) increased, with 162 ms and 202 ms being the only conditions significantly higher than the control condition (No Gap). 1B demonstrates that for all conditions, the presence of gating mechanism (PPIPSI) was significantly higher than the control condition (No Gap)

The GLMM analysis revealed a statistically significant main effect of SOA (*X*^*2*^(5, *N* = 25) = 103, *p* = .0001*). The pattern of results depicted in Fig. 1A showed that the proportion of trials where the ‘pulse with prepulse’ was perceived less intense than the ‘pulse alone’ increased with SOA. On the control condition (No Gap), participants performed at chance, reporting on average that ~49% of ‘pulse with prepulse’ was less intense (*M* = 0.49, *SE* = 0.04). Interestingly, at a 42-ms SOA, there was a statistically significant bias towards facilitation – reporting on average ~40% of trials that the ‘with prepulse’ was less intense (*M* = 0.40, *SE* = .04), meaning ~60% of trials ‘with prepulse’ was perceived more intense – relative to No Gap (*z*(5) = 2.86, *p* < .05). At 82- and 122-ms SOAs, participant responses returned to chance-levels (SOA 82 ms, *M* = 0.52, *SE* = 0.04; SOA 122 ms, *M* = 0.55, *SE* = 0.04) and were not statistically significantly different compared with No Gap (82 vs. No Gap; *p* > .05; 122 vs. No Gap; *p* > .05). At 162 and 202 ms SOA, a statistically significant perceptual bias emerged towards prepulse inhibition – reporting on ~62% and ~69% of trials that ‘pulse with prepulse’ was less intense compared to ‘pulse alone’ (SOA 162 ms: *M* = 0.62, *SE* = 0.04; SOA 202 ms: *M* = 0.69, *SE* = 0.04) – relative to No Gap (162 vs. No Gap: *z*(5) = -3.92, *p* < .001; 200 vs. No Gap: *z*(5) = -6.24, *p* < .001). Pairwise comparisons between SOA 162 ms and SOA 202 ms did not reveal a statistically significant differences in perceptual bias (*z*(5) = -2.48, *p* = 0.07).

### Experiment 2: 202- to 602-ms SOAs

The GLMM analysis revealed a statistically significant main effect of SOA (*X*^*2*^(5, *N* = 23) = 38.7, *p* = .0001*). On the control condition (No Gap), participants performed close to chance (*M* = .47, *SE* = .04). As depicted in Fig. 1B, the results show a consistent pattern of perceptual bias towards prepulse inhibition at 202- to 502-ms SOAs with average reports that on ~63–66% of trials the ‘pulse with prepulse’ was less intense (SOA 202 ms: *M(SE)* = 0.64(0.04); SOA 302 ms = 0.63(0.04); SOA 402 ms = 0.66(0.04); SOA 502 ms = *0*.63(0.04)) with a slight decrease in bias at 602ms (*M* = .58, *SE* = .04). Follow-up pairwise comparisons revealed that the response bias towards prepulse inhibition for all SOAs was significantly greater compared to the No Gap condition (all *p* < .01). Although a slight decrease at 602-ms SOA can be observed in Fig. 1B, no statistically significant difference in response bias was observed between any SOA pair (all *p* > .15).

### Experiment 3: Prepulse intensity (1×, 2× and 3× perceptual threshold)

**Fig. 2:**
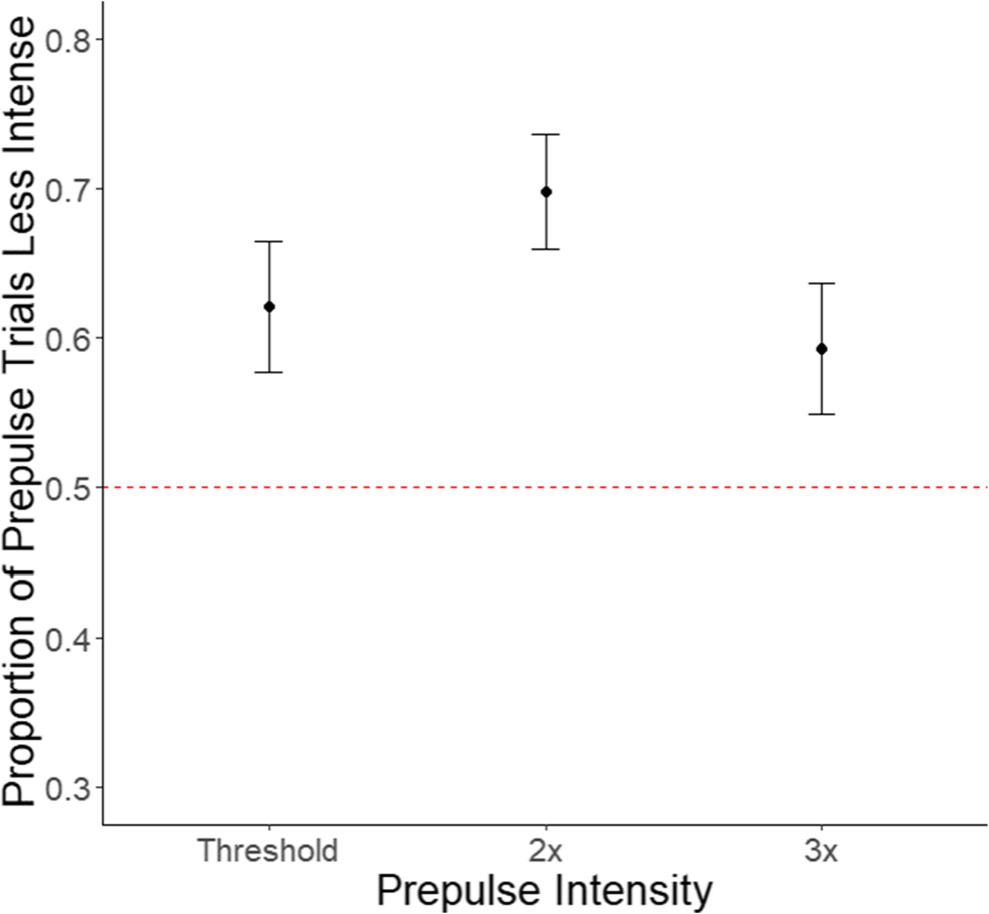
Estimated marginal mean proportion of trials perceived less intense for each prepulse intensity investigated in Experiment 3. The red dotted line represents chance level. Bars represent the SE for each condition. The graph shows that the proportion of PPIPSI was significantly higher in the 2× threshold condition compared to 1× and 3× conditions, where no significant difference was found between 1× and 3× perceptual threshold conditions

The GLMM analysis revealed a statistically significant main effect of prepulse intensity on PPIPSI (*X*^*2*^(2, *N* = 24) = 15.9, *p* = .0003*). As illustrated in Fig. 2, the proportion of ‘pulse with prepulse’ trial reported as less intense was maximal when prepulse intensity was set to 2× the perceptual threshold (*M* = .70, *SE* = .04). Follow-up pairwise comparisons revealed this was significantly greater compared to 1× and 3× perceptual threshold conditions (1×: *M* = .62, *SE* = .04; 1× vs. 2×: (*z*(2) = -2.77, *p* = 0.01; 2× vs. 3×: *M* = .59, *SE* = .04; (*z*(2) = -3.88, *p* = 0.0003). No statistically significant difference in the proportion of PPIPSI between 1× and 3× threshold conditions was observed (*z*(2) = 0.96, *p* = 0.34).

## Discussion

In the current study, we sought to characterise the nature of PPIPSI by examining how it is modified as a function of SOA and prepulse intensity. With respect to timing, we predicted that PPIPSI would not be observed at shorter SOAs and would emerge after ~100 ms, reflecting the dependence of PPIPSI on the reorientation of attention. In Experiment 1, no PPIPSI was observed in the No Gap condition. Interestingly, the prepulse led to an increase in perceived intensity at 42 ms, which could reflect a form of priming or summation (Neumann et al., 2004), followed by a gradual shift towards inhibition with increasing SOA. PPIPSI was observed in ~62–69% of trials at 162 and 202 ms. In Experiment 2, we investigated whether the proportion of PPIPSI would increase beyond SOAs of 202 ms. We observed that the proportion of trials with PPIPSI remained consistent between 202 and 602 ms (~58–66%).

With respect to prepulse intensity, we predicted that the proportion of trials where PPIPSI is observed would increase with prepulse intensity – as this may facilitate the re-orientation of attention towards the pulse. We focused on an SOA of 202 ms when exploring the effect of intensity as PPIPSI was most prominent at this timing (~64–69%). Although Experiment 2 showed that PPIPSI was comparable between 202 and 602 ms, focusing on 202 ms limits the potential influence of voluntary processes that may be evident at longer SOAs. In this last experiment, a prepulse 2 times (2×) stronger than the perceptual threshold elicited the greatest proportion of PPIPSI (~70%), compared to perceptual threshold (1×) and 3× perceptual threshold (~59–62%).

### PPIPSI at SOA > 162 ms suggests the involvement of attentional processes

Collectively, our findings align with conceptualisations of PPIPSI being reliant on attentional and self-monitoring mechanisms – which require greater time between the prepulse and pulse to take effect. Swerdlow et al. (2005) found that the magnitude of perceived intensity reduction increased with SOA; we too found that the proportion of trials where PPIPSI was observed increased with SOA. In their paradigm assessment, Swerdlow et al. (2005) studied the time-course of acoustic PPIPSI at SOAs of 10, 20, 30, 60 and 120 ms – finding that PPIPSI only emerges and is maximal at intervals susceptible to attentional control (approximate reduction: 25% at 120 ms), the attentional control range being 120 ms and above (Dawson et al., 1993). Consistent with these findings, we did not observe a significant proportion of electrotactile PPIPSI until SOAs were within the suggested attentional range (~62% at 162–602 ms), providing further evidence that PPIPSI requires the engagement of attentionally sensitive mechanisms that are not required for startle PPI (Swerdlow et al., 2005; Swerdlow et al., 2007).

#### Mechanism of perceived intensity reduction by prepulses

While the time-course and experimental design of PPIPSI indicate the involvement of attentional and higher cognitive evaluative mechanisms, there are a few ways by which the prepulse might influence these mechanisms, leading to perceived intensity reductions. These include mechanisms of prepulse inhibition and perceptual assimilation. In startle PPI, studies show that the prepulse activation (depending on its modality) travels through the inferior and superior colliculus to the pedunculopontine tegmental nucleus (PPTg), where it results in suppression of the primary startle pathway (for detailed reviews, see Azzopardi et al., 2018; Fendt et al., 2001). Given the strong correlation found between startle PPI and PPIPSI (Swerdlow et al., 1999), the inhibitory effect of PPTg activation by the prepulse may also project to higher processing areas, resulting in inhibited processing of subsequent stimuli. Similarly, though via a different pathway, electrotactile prepulse may activate the lower pain gate in the spinal cord, which then limits the projection of intensity information for the following pulse stimulus, resulting in reduced perceived intensity. PPIPSI may be observed at longer SOAs than startle PPI because more time is required to direct attention (even if driven indirectly by the prepulse) to monitor inputs to the cortex. Note that although participants are likely attending to the somatosensory channel throughout the entire trial, it is difficult to maintain a high level of attention when the exact timing of the stimuli is random (see Bendixen et al., 2009). Therefore, in addition to inhibiting the input to the cortex, the prepulse might serve as a temporal cue to allocate additional resources to monitor particular sensory channels. This model is consistent with Brunia’s (1993) proposal that motor and attention processes use similar mechanisms. More precisely, because motor responses cannot be held in a high state of preparation for long periods (100–300 ms; Alegria, 1975; Müller-Gethmann et al., 2003), responses are maximally prepared when the warning signal is presented around 200 ms before the imperative stimulus to act. In PPIPSI, the participants similarly cannot stay in a high state of attention to perceive the pulse, because the timing of pulse presentation is uncertain.

PPIPSI resembles phenomena known as ‘loudness enhancement’ and ‘loudness decrement’, though these are observed in considerably different procedures (Elmasian et al., 1980). In these experiments, the target stimulus’ perceived loudness is increased when preceded by a louder conditioning stimulus and decreased when preceded by a weaker stimulus (Elmasian et al., 1974; Elmasian et al., 1980; Zwislocki & Ketkar, 1972). The observed effects are said to be due to assimilation, a form of perceptual averaging that occurs due to processing of the two stimuli that are difficult to separate and are overlapping in time (Elmasian et al., 1980). The assimilation process hypothesis holds that assimilation should be maximal when the intensity disparity between the two stimuli is greatest (Elmasian et al., 1980). Our findings that 2× threshold prepulses produce greater PPIPSI are inconsistent with this proposition because the disparity between stimuli was greatest in the 1× threshold condition. This and findings that PPIPSI is observed with cross-modal stimuli (English & Drummond, 2021; Swerdlow et al., 1999) indicate that perceptual averaging is unlikely to provide an explanation for our results.

### The role of stimuli intensity

Although consistent with the broader PPIPSI literature in supporting that longer SOAs provide greater direction of attention to perceive sensory gating effects, several differences between our experiments and these studies offer nuanced insights into PPIPSI, but also limitations.

The broader PPIPSI literature uses fixed stimulus intensities across participants and a visual analogue scale (VAS; assigning a numeric perceived intensity rating) to measure PPIPSI (Swerdlow et al., 1999; Swerdlow et al., 2005; Swerdlow et al., 2007). The VAS method yields a percentage difference value between pulse conditions (with/without prepulse; Swerdlow et al., 2005). However, the use of a percentage reduction method requires a high-intensity pulse-alone stimulus to allow room to observe a significant reduction in perceived intensity in the prepulse-pulse condition. This is evident from Swerdlow et al.’s (2005) calibration session, where 90 dB and 95 dB pulse-alone conditions yielded perceived intensity scores of 10/100 and 20/100, respectively, while the 105 dB pulse alone yielded an approximate score of 80/100. In their case, pulses below 100 dB would have likely been susceptible to a floor effect, limiting the ability to observe a percentage difference in SOAs and subsequently altering PPIPSI’s time-course. An ethical requirement of our study was that stimuli be non-painful; this, in combination with evidence that electrotactile stimuli are more subjectively aversive than acoustic (Sperl et al., 2016), led us to opt for individualised intensities as opposed to predetermined ones. Informed by the evidence from Swerdlow et al.’s (2005) calibration session, a concern of using individualised intensities was that participants may select intensities in ranges below those required to observe meaningful differences between conditions using a percent reduction method. Thus, we selected a comparison between pulse (with/without prepulse) conditions method, which yields a more general proportion of trials perceived as less intense metric.

Using these fixed intensity settings and the VAS, the literature typically finds that PPIPSI increases as prepulse intensity increases to some threshold, after which PPIPSI begins to decrease with further increases in prepulse intensity (Swerdlow et al., 1999; Swerdlow et al., 2007). Despite our use of individualised intensity settings and proportion method, we identified a similar non-linear pattern of prepulse intensity effects to previous studies. We observed an increase in PPIPSI with increased prepulse intensity from 1× to 2×, followed by a reduction in PPIPSI in the 3× perceptual threshold condition.

The literature also suggests PPIPSI is maximal with higher pulse intensities (e.g., Swerdlow et al., 2005). A limitation of our stimulus intensity settings, particularly that the pulse be ‘unpleasant, but not painful’, and our use of a proportion method is that our study is not well equipped to support inferences about pulse intensity effects. Evidence that PPIPSI increases with pulse intensity could be due to something inherent in the gating mechanisms, or a product of the VAS method requiring high intensities to be sensitive (discussed further below). An additional possibility is that the use of proportion may have limited our ability to identify differences in effects between 202 and 602 ms SOAs. The VAS method may reveal that although the proportion of PPIPSI doesn’t change at these intervals, the pattern of percentage reduction between conditions might. To resolve these limitations, a future study may validate the sensitivity of the VAS at lower intensities, and by testing an array of objectively set low to high intensities and array of SOAs.

### Modality-specific differences in PPIPSI

Building on the PPIPSI literature, our study provides evidence of possible modality-specific differences in the time-course of PPIPSI. When compared to acoustic PPIPSI (Swerdlow et al., 2005), our findings suggest that electro-tactile PPIPSI has a longer time-course of activation. Swerdlow et al. (2005), using high-intensity/startling acoustic stimuli (85 dB prepulse/105 dB pulse) identified PPIPSI at 60- and 120-ms SOAs, whereas our findings evidence electrotactile PPIPSI requires SOAs > 162 ms. A possible explanation for this is that like PPI, PPIPSI contains modality-specific processing pathways, for which a faster auditory and slower tactile pathway exist (Gómez-Nieto et al., 2020; Yeomans et al., 2006). This intuitively suggests that faster processing of stimuli reduce the time-course at which attentional mechanisms are recruited and can be directed towards the relevant sensory channels. However, there is evidence to suggest pulse intensity may modulate the time-course of PPIPSI, which may account for this small difference in SOA between our two studies (Swerdlow et al., 2005).

Within the same modality as our study (electro-tactile), though with painful electric shocks (*M* = 160V), Blumenthal et al. (2001) observed PPIPSI at SOAs of 40 and 60 ms using 1× and 1.25× perceptual threshold prepulses. These SOAs are well within the ‘preattentive’ range, and with shorter SOAs than the acoustic modality used by Swerdlow et al. (2005). One possible explanation for these findings is that the high intensity of the pulse in Swerdlow et al.’s (2005) and particularly Blumenthal et al.’s (2001) work may have made participants more sensitive to the effect of the prepulse. That is, in line with the findings that PPIPSI increases with stimuli intensity and the VAS being more sensitive to high-intensity stimuli, the effect of the prepulse may be greater for high-intensity pulses due to the dynamic range of the perceptual system. There is more room for the reduction of a large signal than a small signal. For example, a 20% reduction of 100 is 20, but a 20% reduction of 10 is 2. To gain more conclusive insight into possible modality-specific pathways and the role pulse intensity plays in the time-course of PPIPSI, a future study using a range of pulse intensities (particularly high ones) and the VAS would allow for a more direct comparison.

### Separability of PPIPSI and startle PPI

Due to startle being the index of motor PPI, it is common to use stimuli intensities above or equal to 90 dB for acoustic (Blumenthal, 1999; Swerdlow et al., 1999) and 40 mA for electrotactile startle (Bufacchi, 2017; Sambo et al., 2012b; Sambo et al., 2012a). Notably, acoustic startle has been reported at lower intensities (e.g., 70 dB; Blumenthal & Goode, 1991), though the broader literature suggests it is less reliable, resulting in more trials on which no startle occurs – making motor PPI difficult to measure. In the current study, we show that PPIPSI can be elicited with pulse stimuli as weak as 2 mA, well below that which reliably elicits startle, meaning PPIPSI may be a useful measure of sensory gating where motor responses may interfere or confound data. For example, in Blumenthal et al. (2001), it was difficult to disentangle whether PPIPSI simply reflected the perception of perceived startle response. Our findings also provide tentative evidence that PPIPSI and startle may be separable based on intensity requirements and time-course. A future study could directly look at this relationship using similar non- magnitude estimation methods, as magnitude estimate methods appear to require high intensities to be sensitive (Swerdlow et al., 1999).

Multiple studies have reported evidence of cortical PPI (Dawson et al., 2004;Kedzior et al., 2007 ; San-Martin et al., 2018). When the prepulse is present, the N1 and P2 event related potential (ERP) responses to the pulse are reduced (Dawson et al., 2004; Kedzior et al., 2007; San-Martin et al., 2018). Interestingly, studies measuring startle PPI and cortical PPI simultaneously find weak or no correlation (Kedzior et al., 2007; San-Martin et al., 2018). This may not seem surprising given startle PPI is a motor response typically used as an indicator of subcortical activation (Blumenthal, 2015; Fendt et al., 2001), while N1 and P2 ERPs are products of neural activity that provide information about cortical processing of stimuli (San-Martin et al., 2018). However, given the current and other studies indicating that PPIPSI requires attentional mechanisms, a higher-order process, it may be possible that PPIPSI correlates with these ERPs. For example, enhanced N1 has been attributed to early sensory perception (vigilance) and attention to a stimulus (Mingming et al., 2018; Mishra & Hillyard, 2009). PPIPSI may even correlate stronger with neural PPI than startle PPI, supporting the proposed involvement of higher-order, likely attentional mechanisms.

### Role of attention

Our study and the literature suggest that based on the time-course and design of experiments, PPIPSI requires the engagement of attentional mechanisms to be observed. While stimuli intensity may influence the activation time of attentional mechanisms, strong evidence for their involvement in PPIPSI would be shown by manipulating attention directly. A possible avenue for future research would be to divide attention – by giving participants a secondary task to perform, if attention is crucial to PPIPSI it would be expected that participants perceived intensity of the ‘pulse with prepulse’ condition would be affected in this condition compared to when there is no secondary task.

## Conclusion

In the present study, the parameters that elicited the greatest proportion of trials where the ‘pulse with prepulse’ was perceived less intense were found to be a 2× perceptual threshold prepulse presented at an SOA of 202 ms before the pulse. The current results demonstrate that from 0 ms, except for 42 ms, as SOA increases so does the observation of PPIPSI. Consistent with Swerdlow et al. (2005), we conclude that this relationship supports conceptualisations of PPIPSI requiring attentional and self-monitoring processes – which the longer SOAs allow greater activation of. Our findings also provide evidence that PPIPSI can be elicited using less intense stimuli, something that may be useful for those seeking to investigate the mechanisms involved using physiological measures, such as EEG.

## Data Availability

Published work will have its accompanying data made available via *Dataverse*, *GitHub* or the journals preferred alternative, allowing for secondary analyses by other researchers. The code used to run the experiment (*MATLAB* format) and the data analysis for this submission (*R* format) will also be made available upon request.
